# Bidirectional causality between female reproductive traits and temporomandibular disorders

**DOI:** 10.22514/jofph.2025.058

**Published:** 2025-09-12

**Authors:** Lihan Xu, Xiaofu Yang, Cheng Shu, Yuying Wang, Miao Sun, Mengfei Yu

**Affiliations:** ^1^Stomatology Hospital, School of Stomatology, Zhejiang University School of Medicine, 310006 Hangzhou, Zhejiang, China; ^2^School of Stomatology, Zhejiang University School of Medicine, 310006 Hangzhou, Zhejiang, China; ^3^Key Laboratory of Oral Biomedical Research of Zhejiang Province, 310006 Hangzhou, Zhejiang, China; ^4^Key Laboratory of Organ Development and Regeneration of Zhejiang Province, College of Life and Environmental Science, Hangzhou Normal University, 311121 Hangzhou, Zhejiang, China

**Keywords:** Causal effect, Menarche, Mendelian randomization, Menopause, Reproductive traits, Temporomandibular disorders

## Abstract

**Background**: Temporomandibular disorders (TMD) are common, particularly in females of reproductive age, but it remains unclear if TMD and female reproductive traits directly influence each other. Previous studies have suggested links between reproductive factors, such as the menstrual cycle and menopause, and TMD, yet any causal link is unconfirmed. This study seeks to delineate the reciprocal causal interplay between female reproductive traits and TMD. **Methods**: A bidirectional Mendelian randomization (MR) approach was applied to assess five reproductive traits—age at menarche, first sexual intercourse, first birth, last birth, and menopause—considering TMD as the outcome in one analysis and the exposure in the reverse. Statistical methods, including the inverse variance-weighted method, MR Egger, MR Pleiotropy Residual Sum and Outlier (MR-PRESSO), Cochran’s Q test, and leave-one-out analyses, were used to examine pleiotropy and heterogeneity. **Results**: Later age at first sexual intercourse (odds ratio (OR) = 0.51, 95% confidence intervals (CI) 0.37–0.71, *p* = 6.46 × 10^−5^), first birth (OR = 0.86, 95% CI 0.78–0.95, *p* = 0.003), and last birth (OR = 0.37, 95% CI 0.17–0.78, *p* = 0.009) were identified to be protective against TMD. No significant associations emerged for age at menarche or menopause. However, TMD onset may contribute to delayed menarche (Beta = 0.04, 95% CI 0.01–0.06, *p* = 0.035) without affecting other reproductive traits. **Conclusions**: Genetically determined later timing of first sexual intercourse, first birth, and last birth may protect against TMD, while TMD onset may delay menarche, suggesting a bidirectional relationship between reproductive timing and TMD.

## 1. Introduction

Temporomandibular disorders (TMD) encompass a spectrum of jaw-joint and orofacial irregularities, often involving limited oral mobility, muscle aches and audible clicks or pops in the jaw region [1]. Approximately 5–12% of adults are affected, with a notably higher incidence among younger populations and in women—particularly those of reproductive age—who experience TMD at roughly four times the rate observed in men [2, 3, 4]. Moreover, the United States bears an annual economic burden exceeding 100 billion dollars solely attributable to TMD [5, 6]. Consequently, a thorough investigation into the etiology of TMD and the identification of gender-specific risk factors in females are imperative.

The etiology of TMD is complex, involving biological, environmental, social, emotional, and cognitive triggers frequently cited as contributing factors [7]. Recently, there has been increased interest in understanding how women’s reproductive traits may affect the incidence of TMD [8, 9, 10, 11, 12]. Current research indicates associations between the menstrual cycle [12], pregnancy [8] and menopause [9, 11] with the onset and severity of TMD symptoms. Although these findings suggest a connection between reproductive factors and TMD, the causal relationships remain unclear.

Mendelian randomization (MR) constitutes a causal inference methodology employing germline genetic variants as natural experiments, where single-nucleotide polymorphisms (SNPs) meeting genome-wide significance thresholds serve as instrumental variables (IVs) to estimate etiological effects between modifiable exposures and clinical endpoints through two-sample variance-weighted estimators [13, 14]. Traditional observational studies often struggle to establish causality due to confounding influences, reverse causation, and measurement inconsistencies, even when statistical correlations exist. MR offers an alternative framework to address these limitations by leveraging genetic predisposition as a proxy for exposure, thereby strengthening causal inference.

Despite its potential, no existing bidirectional MR investigation has examined how female reproductive traits may be linked to TMD, nor has any randomized controlled trial specifically targeted this reciprocal relationship. Accordingly, the objective of this study is to explore the causal relationship between women’s reproductive timeline traits and TMD using a bidirectional MR design.

## 2. Material and methods

### 2.1 Investigation design

Our investigation harnessed SNPs sourced from aggregated genome-wide association studies (GWAS) meta-registers as IVs. IVs must satisfy three fundamental conditions: exhibit a robust association with the exposure, remain independent of potential confounding factors, and influence the outcome exclusively through the exposure pathway [15]. The investigation was structured in two phases. Initially, the potential causal link between female reproductive parameters and TMD was explored. Subsequently, the analysis was reversed to evaluate whether a genetic predisposition for TMD correlates with variations in reproductive characteristics.

### 2.2 GWAS summary statistics on women’s reproductive traits and TMD

GWAS summary datasets for five female reproductive metrics—age at menarche (AAM), age at first sexual intercourse (AFS), age at first live birth (AFB), age at last live birth (ALB), and age at natural menopause (ANM)—were harnessed from prominent genetic consortia accessible through public databases such as PubMed and Integrative Epidemiology Unit (IEU) platform (Table 1).

**Table 1.** Genomic repositories utilized in the MR study.

Phenotype	ID	Sample size	Number of SNPs	Type of trait
Age at menarche	ebi-a-GCST90029036	279,470	11,971,701	Continuous
Age at first sexual intercourse	ebi-a-GCST90000045	214,547	16,426,473	Continuous
Age at first live birth	ebi-a-GCST90000048	418,758	10,766,720	Continuous
Age at last live birth	ukb-b-8727	170,248	9,851,867	Continuous
Age at natural menopause	ukb-b-17422	143,819	9,851,867	Continuous
Temporomandibular disorders	finngen_R10_TEMPOROMANDIB	228,812 (6314 cases and 222,498 controls)	21,298,670	Binary

*SNPs: single-nucleotide polymorphisms; ID: identity document.*

GWAS data for AAM were obtained from publicly available data provided by Po-Ru Loh, comprising 279,470 women of European descent. The largest meta-analysis for AFB was carried out by Mills *et al*. [16], integrating information from 36 European studies and involving 418,758 women, with AFB treated as a continuous measure among mothers. For AFS, genetic variants were identified in an extensive GWAS comprising 214,547 European females, with data drawn from the UK Biobank. Additionally, data for ALB and ANM were derived from records provided by Ben Elsworth, encompassing 170,248 and 143,819 individuals of European ancestry, respectively.

To avoid duplication, as the majority of GWAS data for female reproductive traits derive from the UK Biobank, the TMD dataset was sourced from the recent FinnGen project [17]. TMD cases in FinnGen (R10, December 2023) were identified using International Classification of Diseases (ICD) 10 code K07.6, which includes conditions such as Costen syndrome, temporomandibular joint derangement, snapping jaw, and temporomandibular joint-pain-dysfunction syndrome [18]. All diagnoses were sourced from nationwide electronic health records (hospital and specialist clinic registries), in accordance with FinnGen’s endpoint definition. Notably, individuals with jaw dislocation, sprain or chronic pain in the back, neck, head, or limbs were excluded from the control group. The R10 dataset included 6314 TMD cases and 222,498 controls, with 5067 females in the TMD case group.

### 2.3 Screening for IVs

A stringent selection process was implemented to identify appropriate genetic variants as instrumental proxies for both exposures and outcomes. First, we screened the available GWAS for each exposure trait to find SNPs that exhibited a robust association with the exposure, defined as reaching the conventional genome-wide significance threshold of *p* < 5 × 10^−8^. To ensure that selected variants were independent and not in linkage disequilibrium (LD) with each other, we applied a clumping procedure with parameters set at *r*^2^ < 0.001 and a clumping distance of 10,000 kb. This process retained the most strongly associated variant within each LD block and removed all others in LD with it, thereby ensuring the independence of selected instruments. Next, any variants significantly associated with the outcomes (*p* < 5 × 10^−8^) were excluded. To further ensure robust exposure associations, only variants with an *F* statistic greater than 10 were retained—calculated as *F* = *R*^2^/(1 − *R*^2^) × (N − K − 1)/K, where *R*^2^ was defined as *R*^2^ = 2 × minor allele frequency (MAF) × (1 − MAF) × Beta^2^. Furthermore, reverse-complementary variants displaying uncertain allele distributions were excluded to ensure a uniform effect direction. Finally, to secure a sufficient number of proxies for TMD, variants meeting a genome-wide significance threshold of *p* < 5 × 10^−6^ were included.

### 2.4 Statistical analysis

Causality was chiefly determined by employing the inverse variance weighted (IVW) technique. Complementary analyses incorporated both the MR Egger and weighted median (WM) approaches. To examine heterogeneity, Cochran’s Q (from the IVW model) and Rucker’s Q (from the MR Egger approach) were applied. Detection of horizontal pleiotropy was achieved through the MR Egger intercept test and a global assessment via MR-PRESSO, which also features a distortion test to determine the influence of outliers. When outliers were identified, the analysis was repeated following their removal. The overall stability of the findings was further examined using a leave-one-out sensitivity analysis. All computations were executed with the TwoSampleMR package in R (version 4.3.1). To adjust for multiple comparisons, a Bonferroni-corrected significance level of *p* < 0.01 (*i.e.*, *p* < 0.05/5) was applied, with *p*-values between 0.01 and 0.05 regarded as suggestive.

## 3. Results

### 3.1 Screening for IVs

Following a rigorous selection process to pinpoint exposure-related SNPs and eliminate those in linkage disequilibrium, a total of 243, 73, 65, 6, 114 and 15 SNPs were retained for AAM, AFS, AFB, ALB, ANM and TMD, respectively. Aside from the snoring variant, all selected instruments exhibited *F*-statistic values greater than 10, thereby satisfying the strong relevance requirement for MR analyses. **Supplementary material** provides detailed information about all SNPs.

For the female reproductive exposures, SNP filtering was performed as follows. For AAM, one SNP was missing in the TMD dataset and 11 palindromic variants (rs12926791, rs1327942, rs17089911, rs1874984, rs2725405, rs35999162, rs4818008, rs566465, rs57149692, rs815715, rs9945304) were excluded, leaving 231 SNPs for analysis. For AFS, 4 SNPs were absent and 7 palindromic variants (rs11076963, rs11627661, rs1226414, rs12541633, rs62166484, rs66632973, rs7942078) were removed, resulting in 62 SNPs. In the case of AFB, 3 SNPs were not present and 11 palindromic SNPs (rs10111950, rs10445366, rs13319205, rs1590949, rs1950404, rs3096695, rs4799950, rs6079581, rs7931884, rs7958796, rs9814726) were discarded, yielding 51 SNPs. No palindromic variants were identified for ALB. For ANM, 4 SNPs were absent and 4 palindromic variants (rs1020622, rs394448, rs728900, rs8045589) were eliminated, leaving 106 SNPs for further analysis.

When TMD served as the exposure, the selection process differed by outcome. For AAM, ALB and ANM, one palindromic variant (rs1811546) was detected, yielding a final set of 14 SNPs. In contrast, for AFS and AFB, one SNP was missing from the summary data, and two palindromic variants (rs117730127 and rs1811546) were excluded, resulting in 12 SNPs being retained for analysis.

### 3.2 Causal impact of women’s reproductive traits on TMD

Table 2 presents the outcomes of the MR investigation, where female reproductive parameters were treated as the exposures and TMD as the endpoint.

**Table 2.** The causal effects of women’s reproductive traits on temporomandibular disorders.

Exposure	Outcome	nSNP	Methods	OR (95% CI)	*p*-value	Heterogeneity		Pleiotropy *p*val
						MR Egger Q_*p*val	IVW Q_*p*val	
		231	IVW	1.03 (0.95–1.11)	0.47			
Age at menarche	Temporomandibular disorders	231	MR Egger	1.05 (0.86–1.29)	0.606	**0.003**	**0.003**	0.790
		231	WM	1.02 (0.90–1.14)	0.791			
		62	IVW	0.51 (0.37–0.71)	**6.46 × 10^−5^**			
Age at first sexual intercourse	Temporomandibular disorders	62	MR Egger	0.67 (0.14–3.25)	0.622	**0.002**	**0.002**	0.734
		62	WM	0.60 (0.41–0.88)	**0.009**			
		51	IVW	0.86 (0.78–0.95)	**0.003**			
Age at first sexual intercourse	Temporomandibular disorders	51	MR Egger	0.76 (0.48–1.21)	0.258	**0.009**	**0.011**	0.593
		51	WM	0.86 (0.75–0.97)	**0.016**			
		6	IVW	0.37 (0.17–0.78)	**0.009**			
Age at last live birth	Temporomandibular disorders	6	MR Egger	1.07 (0.04–28.22)	0.971	0.829	0.860	0.546
		6	WM	0.44 (0.17–1.14)	0.091			
		106	IVW	0.99 (0.88–1.11)	0.899			
Age at natural menopause	Temporomandibular disorders	106	MR Egger	1.10 (0.85–1.41)	0.476	0.061	0.062	0.387
		106	WM	1.01 (0.85–1.19)	0.952			

*IVW: Inverse variance weighted; WM: Weighted median; MR: Mendelian randomization; nSNP: number of single nucleotide polymorphisms; OR: odds ratio; CI: confidence intervals; Q_pval: Cochran’s Q-test p value; pval: p value. Bold format represents p < 0.05 considered statistically different.*

Regarding AAM, none of the three analytical approaches—IVW (OR = 1.03, 95% CI 0.95–1.11, *p* = 0.470), MR Egger (OR = 1.05, 95% CI 0.86–1.29, *p* = 0.606), or weighted median (OR = 1.02, 95% CI 0.90–1.14, *p* = 0.791)—revealed a significant association with TMD risk. Although heterogeneity was initially detected, the exclusion of outlier SNPs (rs12926791, rs1327942, rs17089911, rs1874984, rs2725405, rs35999162, rs4818008, rs566465, rs57149692, rs815715, rs9945304) did not change the null findings. Heterogeneity was identified in the inconsistency analysis. After eliminating the origin of heterogeneity (rs12926791, rs1327942, rs17089911, rs1874984, rs2725405, rs35999162, rs4818008, rs566465, rs57149692, rs815715, rs9945304), AAM continued to exhibit no association to TMD (**Supplementary Table 1**). Additionally, the analysis did not uncover evidence of horizontal pleiotropy. The corresponding sensitivity assessment framework was illustrated in **Supplementary Fig. 1**.

Regarding AFS, both the IVW and WM methods showed that a later AFS conferred a protective effect against TMD (IVW: OR = 0.51, 95% CI 0.37–0.71, *p* = 6.46 × 10^−5^; WM: OR = 0.60, 95% CI 0.41–0.88, *p* = 0.009). In contrast, the MR Egger approach did not yield a statistically significant association (OR = 0.67, 95% CI 0.14–3.25, *p* = 0.622). Although initial tests detected heterogeneity, the exclusion of outlier SNPs (rs11076963, rs11627661, rs1226414, rs12541633, rs62166484, rs66632973, rs7942078) did not alter the results (**Supplementary Table 1**). Additionally, no horizontal pleiotropy was observed, and leave-one-out sensitivity analyses confirmed that the overall IVW estimate remained consistent when any single SNP was omitted (Fig. 1).

**Fig. 1.** Leave-one-out plots, funnel plots, and scatter plots for AFS as exposure and TMD as outcome. MR: Mendelian randomization; SNP: single-nucleotide polymorphism; SE_IV_: standard error of instrumental variable estimate; β_IV_: instrumental variable estimate of the causal effect.

Regarding AFB, both the IVW and WM analyses revealed that a later AFB was linked to a diminished susceptibility to TMD (IVW: OR = 0.86, 95% CI 0.78–0.95, *p* = 0.003; WM: OR = 0.86, 95% CI 0.75–0.97, *p* = 0.016). In contrast, the MR Egger model did not yield a statistically significant estimate (OR = 0.76, 95% CI 0.48–1.21, *p* = 0.258). Although initial heterogeneity was detected, removing outlier SNPs (rs10111950, rs10445366, rs13319205, rs1590949, rs1950404, rs3096695, rs4799950, rs6079581, rs7931884, rs7958796, rs9814726) did not change the overall findings (**Supplementary Table 1**). Additionally, no horizontal pleiotropy was detected, and sensitivity tests confirmed that removing any single SNP did not materially affect the overall estimate (Fig. 2).

**Fig. 2.** Leave-one-out plots, funnel plots, and scatter plots for AFB as exposure and TMD as outcome. MR: Mendelian randomization; SNP: single-nucleotide polymorphism; SE_IV_: standard error of instrumental variable estimate; β_IV_: instrumental variable estimate of the causal effect.

Regarding ALB, the IVW approach revealed that a later age at last live birth significantly reduced the risk of TMD (OR = 0.37, 95% CI 0.17–0.78, *p* = 0.009). In contrast, neither the MR Egger (OR = 1.07, 95% CI 0.04–28.22, *p* = 0.971) nor the WM methods (OR = 0.44, 95% CI 0.17–1.14, *p* = 0.091) produced statistically significant results. There was no indication of heterogeneity or horizontal pleiotropy, and the sensitivity test confirmed the stability of these findings (Fig. 3).

**Fig. 3.** Leave-one-out plots, funnel plots, and scatter plots for ALB as exposure and TMD as outcome. MR: Mendelian randomization; SNP: single-nucleotide polymorphism; SE_IV_: standard error of instrumental variable estimate; β_IV_: instrumental variable estimate of the causal effect.

Regarding AAM, all three analytical strategies consistently showed that genetically determined ANM bears no association with TMD risk (IVW: OR = 0.99, 95% CI 0.88–1.11, *p* = 0.899; MR Egger: OR = 1.10, 95% CI 0.85–1.41, *p* = 0.476; WM: OR = 1.01, 95% CI 0.85–1.19, *p* = 0.952). No heterogeneity or evidence of unintended genetic effects was observed, and additional diagnostic visualizations are provided in **Supplementary Fig. 2**.

### 3.3 Causal impact of TMD on women’s reproductive traits

Table 3 summarizes the MR findings, with TMD serving as the exposure variable and female reproductive traits as the outcomes. The IVW approach revealed no overall effect of TMD on these reproductive markers. However, heterogeneity was noted for AAM and ANM. Once the sources of variability were removed (**Supplementary Table 2**), the analysis indicated that TMD may delay AAM (IVW: Beta = 0.04, 95% CI 0.01–0.06, *p* = 0.035), while no significant association was detected for ANM (IVW: Beta = 0.02, 95% CI −0.01–0.04, *p* = 0.137). **Supplementary Figs. 3,4,5,6,7** provide further details through leave-one-out, funnel and scatter plots.

**Table 3.** The causal effects of temporomandibular disorders on women’s reproductive traits.

Exposure	Outcome	nSNP	Methods	OR/Beta (95% CI)	*p*-value	Heterogeneity		Pleiotropy *p*-val
						MR Egger Q_*p*val	IVW Q_*p*val	
		14	IVW	0.02 (−0.01–0.06)	0.195			
Temporomandibular disorders	Age at menarche	14	MR Egger	0.03 (−0.02–0.09)	0.260	**0.001**	**0.001**	0.628
		14	WM	0.03 (0.00–0.06)	**0.047**			
		12	IVW	0.01 (−0.01–0.02)	0.196			
Temporomandibular disorders	Age at first sexual intercourse	12	MR Egger	0.01 (−0.02–0.03)	0.599	0.868	0.907	0.735
		12	WM	0.01 (−0.01–0.03)	0.480			
		12	IVW	−0.04 (−0.11–0.02)	0.195			
Temporomandibular disorders	Age at first sexual intercourse	12	MR Egger	0.03 (−0.07–0.13)	0.609	0.310	0.176	0.113
		12	WM	−0.01 (−0.09–0.07)	0.871			
		14	IVW	0.00 (−0.02–0.02)	0.960			
Temporomandibular disorders	Age at last live birth	14	MR Egger	0.01 (−0.01–0.04)	0.380	0.792	0.758	0.278
		14	WM	0.00 (−0.02–0.03)	0.913			
		14	IVW	0.01 (−0.02–0.04)	0.436			
Temporomandibular disorders	Age at natural menopause	14	MR Egger	0.05 (0.01–0.08)	**0.026**	0.068	**0.004**	**0.027**
		14	WM	0.02 (−0.01–0.05)	0.100			

*IVW: Inverse variance weighted; WM: Weighted median; MR: Mendelian randomization; nSNP: number of single nucleotide polymorphisms; OR: odds ratio; CI: confidence intervals; Q_pval: Cochran’s Q-test p value; p-val: p value. Bold format represents p < 0.05 considered statistically different.*

## 4. Discussion

This investigation marked the inaugural application of MR to probe the genetic-level causal associations between female reproductive traits and TMD. Extensive genomic data from European cohorts were utilized to evaluate several reproductive markers—specifically, AAM, AFS, AFB, ALB, and ANM—to uncover potential causal relationships with TMD. The findings demonstrated that genetically predicted later AFS, AFB and ALB were linked to a reduced risk of TMD. Notably, each additional year delay in AFS correlated with an approximately 49% reduction in the likelihood of developing TMD (OR: 0.51; 95% CI: 0.37–0.71), highlighting a substantial protective benefit. Similarly, postponing AFB by one year was associated with a moderate but meaningful 14% reduction in TMD risk (OR: 0.86; 95% CI: 0.78–0.95), while delaying ALB demonstrated an even stronger protective effect, associated with roughly a 63% lower risk (OR: 0.37; 95% CI: 0.17–0.78). For the reverse direction, TMD appeared to delay the onset of menarche, while other reproductive characteristics remained unaffected. For example, a genetic predisposition to TMD slightly delayed the onset of menarche by about 0.04 years (approximately two weeks). This effect size is small, implying minimal clinical impact on an individual’s pubertal timing. Overall, our results suggest a bidirectional link between certain reproductive traits and TMD: later reproductive events confer protection against TMD, and TMD in turn may slightly influence the timing of menarche. Our findings carry several implications for clinical and public health practice regarding TMD in women. Recognizing reproductive history as a factor influencing TMD risk may help clinicians better stratify risk and potentially tailor preventive or early intervention strategies for women with early reproductive milestones.

Menstruation and childbirth represent significant milestones in a woman’s life. Prior research on women’s reproductive traits has identified causal links between female reproduction and psychiatric disorders, ovarian cysts, breast cancer and other diseases [19, 20, 21, 22, 23]. In this study, we found no evidence of a causal link between AAM and TMD, aligning with a large cross-sectional study that similarly reported no significant association between a girl’s age at first menstruation and her likelihood of developing TMD later in life [24]. Our findings suggest that, although the reverse causal effect of TMD on AAM is minimal, this subtle influence hints at an intriguing biological connection: genetic factors predisposing an individual to TMD may intersect with the pathways regulating pubertal timing. Previous research indicates that chronic illness or malnutrition during adolescence can delay menarche [25]. TMD may impair chewing function [26], which in turn can affect nutritional intake [27]. Additionally, we observed no correlation between ANM and the onset of TMD. Nonetheless, prior studies have suggested a higher TMD prevalence among postmenopausal women relative to premenopausal counterparts, with postmenopausal TMD patients typically experiencing more severe symptoms [9, 10]. Discrepancies in findings might stem from cross-sectional study limitations, which cannot fully rule out confounding or age-related biases. Postmenopausal women are generally older, and age or related health factors could explain higher TMD rates. Conversely, our study utilized the MR approach, revealing no elevated TMD risk associated with ANM.

Moreover, our investigation revealed that AFB and ALB serve as protective factors against TMD. In other words, women who delay childbirth may experience a decreased likelihood of TMD development. While the precise mechanisms remain elusive, hormonal fluctuations, particularly alterations in estrogen and progesterone levels during pregnancy, could be contributing factors. The association between estrogen levels and TMD remains contentious, with potential discrepancies in the impacts of endogenous and exogenous estrogen. Two studies propose a positive correlation between elevated estrogen levels and TMD prevalence [28, 29]. Endogenous estrogen may influence the temporomandibular joint’s bone, cartilage, and associated structures, potentially causing internal derangements [30, 31, 32]. Furthermore, this hormone can trigger distinct inflammatory reactions within the joint [33]. Exogenous estrogen use may potentially elevate the risk of TMD [34]. Nevertheless, certain studies suggest that estrogen replacement therapy does not heighten the risk of TMD development [32, 35]. These discrepancies may relate to varying estrogen levels: perimenopausal or postmenopausal women generally start from a lower baseline, so even with supplemental estrogen, their levels might remain within a “safe” threshold for TMD. Thus, excessive estrogen levels indeed elevate the risk of TMD. This impact of childbirth on TMD might stem from the higher estrogen levels typically seen in younger individuals compared to older ones. An animal study suggests that the peak levels of estradiol in aged pregnant macaques during pregnancy are lower than those in younger pregnant macaques, indicating that the increase in estrogen levels during pregnancy is restricted in older mothers [36]. Pregnancy exacerbates estrogen levels, yet when pregnancy occurs later in life, although estrogen levels rise, they stay within a safe threshold. This milder hormonal environment may reduce the risk of TMD triggered by pregnancy. Overall, delaying pregnancy is considered to potentially help reduce the adverse effects of dramatic hormonal fluctuations on the temporomandibular joint. Moreover, progesterone, a hormone that substantially rises during pregnancy, might confer a protective effect against TMD. Although some studies reported no disparity in progesterone levels between TMD patients and controls, two larger studies suggested lower progesterone levels in TMD patients [28, 37, 38]. Progesterone’s protective effect against TMD may stem from its capacity to ameliorate temporomandibular joint inflammation by inhibiting the Nuclear factor kappa-light-chain-enhancer of activated B cells (NF-κB) pathway and modulating the trigeminal ganglion Nav1.7 [39, 40]. Progesterone supplementation can alleviate TMD pain [41, 42, 43]. Correspondingly, as women age, the levels of progesterone in their bodies start to decrease. Therefore, a woman who becomes pregnant later in life might benefit from the anti-inflammatory effects of progesterone at an age when her estrogen levels are relatively tempered, collectively contributing to a lower risk of TMD. These hormonal explanations remain speculative, as TMD pathophysiology is multifactorial and demands further research to clarify the precise mechanisms. Nonetheless, the protective causal effect of later AFB and ALB observed in our study provides a new piece of evidence supporting the idea that the timing of reproductive events can influence a woman’s musculoskeletal health in the jaw region.

Beyond biological mechanisms, there may also be social or behavioral factors tied to reproductive timing that impact TMD risk. Women who have children earlier might experience different stress levels and lifestyle patterns—such as sleep disruption or nutritional challenges during early motherhood—compared to those who give birth later [44, 45]. Chronic stress, whether physical or emotional, can exacerbate muscle tension and pain, thereby contributing to TMD [46, 47]. Therefore, some risks associated with early childbirth or early sexual activity may be mediated by these socio-psychological factors. However, since our MR design accounts for many confounding factors, the direct genetic evidence for causality suggests that, even if these mediators are at play, reproductive timing itself remains an independent factor in the etiology of TMD.

Women in their childbearing years face a markedly elevated risk of developing TMD compared to other age groups and men. Thus, they must take preventive measures against TMD. Doing so can potentially reduce the additional time and financial burden often associated with treatment. Our findings indicate that postponing sexual intercourse and childbirth may decrease the risk of TMD development. In other words, women who engage in sexual intercourse and childbearing at younger ages should be vigilant about preventing TMD.

This investigation offers several notable advantages. First, its internal validity is primarily attributable to the use of MR design, a widely adopted statistical method that leverages randomly allocated genetic variants (SNPs) at conception to approximate a natural randomization process [48, 49, 50, 51, 52]. Since genetic variants are generally unaffected by subsequent lifestyle or environmental factors, MR significantly reduces confounding arising from socioeconomic status, diet, and healthcare behaviors, which often complicate observational studies [53]. Moreover, MR effectively addresses reverse causation, as genetic variants are established before disease onset, ensuring that any link between reproductive traits and TMD reflect genuine causal relationships rather than the reverse. Additionally, we conducted multiple sensitivity analyses to verify the robustness of our causal estimates and detect any potential pleiotropy or outliers, thereby strengthening the reliability of our inferences. Second, the large sample size and comprehensive dataset on reproductive variables enabled estimate effects that closely approximate reality. We also included five distinct reproductive traits (AAM, AFS, AFB, ALB, and ANM) to comprehensively depict and complement reproductive characteristics. Nonetheless, several methodological considerations warrant attention. Primarily, the aggregated GWAS datasets exclusively comprised European-ancestry cohorts, potentially constraining the applicability of causal estimates to populations with divergent linkage disequilibrium patterns or socioenvironmental contexts. Second, heterogeneity observed in some analyses might introduce bias into the causal estimates; however, leave-one-out evaluations suggested that the risk estimates were consistent. Lastly, the reliance on self-reported reproductive traits raises concerns regarding recall bias and measurement errors, possibly diminishing statistical power to a slight degree.

## 5. Conclusions

This investigation represented the inaugural application of MR analysis to explore the genetic-level links between female reproductive traits and TMD. The results indicated that later AFS, AFB and ALB were associated with a lower risk of developing TMD. Furthermore, TMD appeared to postpone ANM, while other reproductive measures remained unaffected. These effects may be caused by hormonal changes, and future studies are needed to further explore the mediating role of hormones.

## Supplementary Material

Supplementary material associated with this article can be found, in the online version, at https://files.jofph.com/ files/article/1966377393403641856/attachment/ Supplementary%20material.zip.







## References

[1] Durham J, Newton-John TR, Zakrzewska JM. Temporomandibular disorders. The BMJ. 2015; 350: h1154. 10.1136/bmj.h1154 25767130

[2] Qin H, Guo S, Chen X, Liu Y, Lu L, Zhang M, *et al*. Clinical profile in relation to age and gender of patients with temporomandibular disorders: a retrospective study. BMC Oral Health. 2024; 24: 955. 10.1186/s12903-024-04736-2 PMC11330063 39152429

[3] Lövgren A, Vallin S, Häggman-Henrikson B, Kapos FP, Peck CC, Visscher CM, *et al*. Women are worse off in developing and recovering from temporomandibular disorder symptoms. Scientific Reports. 2025; 15: 4732. 10.1038/s41598-025-86502-0 PMC11807177 39922904

[4] Matheson EM, Fermo JD, Blackwelder RS. Temporomandibular disorders: rapid evidence review. American Academy of Family Physicians. 2023; 107: 52–58. 36689971

[5] Jin L, Lamster I, Greenspan J, Pitts N, Scully C, Warnakulasuriya S. Global burden of oral diseases: emerging concepts, management and interplay with systemic health. Oral Diseases. 2016; 22: 609–619. 10.1111/odi.12428 26704694

[6] Lee KS, Jha N, Kim YJ. Risk factor assessments of temporomandibular disorders via machine learning. Scientific Reports. 2021; 11: 19802. 10.1038/s41598-021-98837-5 PMC8492627 34611188

[7] Gauer RL, Semidey MJ. Diagnosis and treatment of temporomandibular disorders. American Academy of Family Physicians. 2015; 91: 378–386. 25822556

[8] Fichera G, Polizzi A, Scapellato S, Palazzo G, Indelicato F. Craniomandibular disorders in pregnant women: an epidemiological survey. Journal of Functional Morphology and Kinesiology. 2020; 5: 36. 10.3390/jfmk5020036 PMC7739292 33467252

[9] Mursu E, Yu J, Karjalainen E, Savukoski S, Niinimäki M, Näpänkangas R, *et al*. Association of climacterium with temporomandibular disorders at the age of 46 years—a cross-sectional study. Acta Odontologica Scandinavica. 2023; 81: 319–324. 10.1080/00016357.2022.2146746 36403169

[10] Farzin M, Taghva M, Babooie M. Comparison of temporomandibular disorders between menopausal and non-menopausal women. Journal of the Korean Association of Oral and Maxillofacial Surgeons. 2018; 44: 232–236. 10.5125/jkaoms.2018.44.5.232 PMC6209692 30402415

[11] Galhardo APM, Mukai MK, Baracat MCP, da Fonseca AM, Roa CL, Sorpreso ICE, *et al*. Does temporomandibular disorder correlate with menopausal symptoms? Menopause. 2022; 29: 728–733. 10.1097/GME.0000000000001962 35544600

[12] Jedynak B, Jaworska-Zaremba M, Grzechocińska B, Chmurska M, Janicka J, Kostrzewa-Janicka J. TMD in females with menstrual disorders. International Journal of Environmental Research and Public Health. 2021; 18: 7263. 10.3390/ijerph18147263 PMC8306893 34299715

[13] Davey Smith G, Ebrahim S. ’Mendelian randomization’: can genetic epidemiology contribute to understanding environmental determinants of disease? International Journal of Epidemiology. 2003; 32: 1–22. 10.1093/ije/dyg070 12689998

[14] Lawlor DA, Harbord RM, Sterne JA, Timpson N, Davey Smith G. Mendelian randomization: using genes as instruments for making causal inferences in epidemiology. Statistics in Medicine. 2008; 27: 1133–1163. 10.1002/sim.3034 17886233

[15] Sekula P, Del Greco M F, Pattaro C, Köttgen A. Mendelian randomization as an approach to assess causality using observational data. Journal of the American Society of Nephrology. 2016; 27: 3253–3265. 10.1681/ASN.2016010098 PMC5084898 27486138

[16] Mills MC, Tropf FC, Brazel DM, van Zuydam N, Vaez A, Pers TH, *et al*. Identification of 371 genetic variants for age at first sex and birth linked to externalising behaviour. Nature Human Behaviour. 2021; 5: 1717–1730. 10.1038/s41562-021-01135-3 PMC7612120 34211149

[17] Kurki MI, Karjalainen J, Palta P, Sipilä TP, Kristiansson K, Donner KM, *et al*. FinnGen provides genetic insights from a well-phenotyped isolated population. Nature. 2023; 613: 508–518. 10.1038/s41586-022-05473-8 PMC9849126 36653562

[18] Zhang L, Zhang Q, Zhao D. Causal links between bone diseases and temporomandibular disorders. International Dental Journal. 2025; 75: 1951–1960. 10.1016/j.identj.2025.01.010 PMC12142797 39880715

[19] Yu Y, Hou L, Wu Y, Yu Y, Liu X, Wu S, *et al*. Causal associations between female reproductive behaviors and psychiatric disorders: a lifecourse Mendelian randomization study. BMC Psychiatry. 2023; 23: 799. 10.1186/s12888-023-05203-y PMC10621101 37915018

[20] Sun W, Wu X, Yang H, Yuan S, Chen J, Fang Y, *et al*. Identifying causal associations between women’s reproductive traits and risk of schizophrenia: a multivariate validated two-sample Mendelian randomization analysis. BMC Psychiatry. 2024; 24: 161. 10.1186/s12888-024-05614-5 PMC10893634 38395764

[21] Su Q, Yang Z. Age at first birth, age at menopause, and risk of ovarian cyst: a two-sample Mendelian randomization study. Frontiers in Endocrinology. 2023; 14: 1279493. 10.3389/fendo.2023.1279493 PMC10794498 38239975

[22] Jia L, Lv W, Liang L, Ma Y, Ma X, Zhang S, *et al*. The causal effect of reproductive factors on breast cancer: a two-sample mendelian randomization study. Journal of Clinical Medicine. 2023; 12: 347. 10.3390/jcm12010347 PMC9820938 36615147

[23] Pang L, Wu K, Su P, Liao Z, Lv C. Mendelian randomization analysis of female reproductive factors on osteoarthritis. Medicine. 2025; 104: e41362. 10.1097/MD.0000000000041362 PMC11789898 39889186

[24] Fernandes G, van Selms MK, Gonçalves DA, Lobbezoo F, Camparis CM. Factors associated with temporomandibular disorders pain in adolescents. Journal of Oral Rehabilitation. 2015; 42: 113–119. 10.1111/joor.12238 25244610

[25] Karapanou O, Papadimitriou A. Determinants of menarche. Reproductive Biology and Endocrinology. 2010; 8: 115. 10.1186/1477-7827-8-115 PMC2958977 20920296

[26] Mélou C, Sixou JL, Sinquin C, Chauvel-Lebret D. Temporomandibular disorders in children and adolescents: a review. Archives de PéDiatrie. 2023; 30: 335–342. 10.1016/j.arcped.2023.03.005 37147156

[27] Kumar A, Almotairy N, Merzo JJ, Wendin K, Rothenberg E, Grigoriadis A, *et al*. Chewing and its influence on swallowing, gastrointestinal and nutrition-related factors: a systematic review. Critical Reviews in Food Science and Nutrition. 2023; 63: 11987–12017. 10.1080/10408398.2022.2098245 35837677

[28] Landi N, Manfredini D, Lombardi I, Casarosa E, Bosco M. 17-beta-estradiol and progesterone serum levels in temporomandibular disorder patients. Minerva Stomatologica. 2004; 53: 651–660. 15894940

[29] Landi N, Lombardi I, Manfredini D, Casarosa E, Biondi K, Gabbanini M, *et al*. Sexual hormone serum levels and temporomandibular disorders. a preliminary study. Gynecological Endocrinology. 2005; 20: 99–103. 10.1080/09513590400021136 15823829

[30] Wang J, Chao Y, Wan Q, Zhu Z. The possible role of estrogen in the incidence of temporomandibular disorders. Medical Hypotheses. 2008; 71: 564–567. 10.1016/j.mehy.2008.05.011 18597950

[31] Kou X, Wu Y, Ding Y, Hao T, Bi R, Gan Y, *et al*. 17β-estradiol aggravates temporomandibular joint inflammation through the NF-κB pathway in ovariectomized rats. Arthritis & Rheumatology. 2011; 63: 1888–1897. 10.1002/art.30334 21391199

[32] Alam MK, Ibrahim MA, Almaslamani MJ, Saeed MH, Siurkel Y, Ronsivalle V, *et al*. Correlating estrogen replacement therapy and temporomandibular disorders: a comprehensive review following PRISMA principles and Cochrane handbook for systematic reviews of interventions. BMC Oral Health. 2024; 24: 93. 10.1186/s12903-023-03697-2 PMC10792960 38229132

[33] Cairns BE. Pathophysiology of TMD pain—basic mechanisms and their implications for pharmacotherapy. Journal of Oral Rehabilitation. 2010; 37: 391–410. 10.1111/j.1365-2842.2010.02074.x 20337865

[34] LeResche L, Saunders K, Von Korff MR, Barlow W, Dworkin SF. Use of exogenous hormones and risk of temporomandibular disorder pain. Pain. 1997; 69: 153–160. 10.1016/s0304-3959(96)03230-7 9060026

[35] Hatch JP, Rugh JD, Sakai S, Saunders MJ. Is use of exogenous estrogen associated with temporomandibular signs and symptoms? The Journal of the American Dental Association. 2001; 132: 319–326. 10.14219/jada.archive.2001.0174 11258088

[36] Plant M, Armstrong C, Ruggiero A, Sherrill C, Uberseder B, Jeffries R, *et al*. Advanced maternal age impacts physiologic adaptations to pregnancy in vervet monkeys. GeroScience. 2020; 42: 1649–1661. 10.1007/s11357-020-00219-8 PMC7732933 32588342

[37] Madani AS, Shamsian AA, Hedayati‐Moghaddam MR, Fathi‐Moghadam F, Sabooni MR, Mirmortazavi A, *et al*. A cross-sectional study of the relationship between serum sexual hormone levels and internal derangement of temporomandibular joint. Journal of Oral Rehabilitation. 2013; 40: 569–573. 10.1111/joor.12074 23710731

[38] Yazici H, Taskin MI, Guney G, Hismiogullari AA, Arslan E, Tulaci KG. The novel relationship between polycystic ovary syndrome and temporomandibular joint disorders. Journal of Stomatology Oral and Maxillofacial Surgery. 2021; 122: 544–548. 10.1016/j.jormas.2020.10.008 33161171

[39] Xue XT, Kou XX, Li CS, Bi RY, Meng Z, Wang XD, *et al*. Progesterone attenuates temporomandibular joint inflammation through inhibition of NF-κB pathway in ovariectomized rats. Scientific Reports. 2017; 7: 15334. 10.1038/s41598-017-15285-w PMC5681685 29127312

[40] Bi RY, Zhang XY, Zhang P, Ding Y, Gan YH. Progesterone attenuates allodynia of inflamed temporomandibular joint through modulating voltage-gated sodium channel 1.7 in trigeminal ganglion. Pain Research and Management. 2020; 2020: 6582586. 10.1155/2020/6582586 PMC7399782 32774568

[41] Hornung RS, Benton WL, Tongkhuya S, Uphouse L, Kramer PR, Averitt DL. Progesterone and allopregnanolone rapidly attenuate estrogen-associated mechanical allodynia in rats with persistent temporomandibular joint inflammation. Frontiers in Integrative Neuroscience. 2020; 14: 26. 10.3389/fnint.2020.00026 PMC7225267 32457584

[42] Hornung RS, Raut NG, Cantu DJ, Lockhart LM, Averitt DL. Sigma-1 receptors and progesterone metabolizing enzymes in nociceptive sensory neurons of the female rat trigeminal ganglia: a neural substrate for the antinociceptive actions of progesterone. Molecular Pain. 2022; 18: 17448069211069255. 10.1177/17448069211069255 PMC8777333 35040378

[43] Leucuța DC, Anton D, Almășan O. Estrogen hormones’ implications on the physiopathology of temporomandibular dysfunction. Journal of Clinical Medicine. 2024; 13: 4406. 10.3390/jcm13154406 PMC11313074 39124673

[44] Yung ST, Main A, Walle EA, Scott RM, Chen Y. Associations between sleep and mental health among Latina adolescent mothers: the role of social support. Frontiers in Psychology. 2021; 12: 647544. 10.3389/fpsyg.2021.647544 PMC8175805 34093329

[45] Flaherty SC, Sadler LS. Parenting stress among adolescent mothers: an integrative literature review. Western Journal of Nursing Research. 2022; 44: 701–719. 10.1177/01939459211014241 PMC9142484 35311420

[46] Lee YH, Auh QS. Clinical factors affecting depression in patients with painful temporomandibular disorders during the COVID-19 pandemic. Scientific Reports. 2022; 12: 14667. 10.1038/s41598-022-18745-0 PMC9421627 36038574

[47] Al-Khudhairy MW, Al-Mutairi A, Al Mazyad B, Al Yousef S, Hatab Alanazi S. The association between post-traumatic stress disorder and temporomandibular disorders: a systematic review. Cureus. 2022; 14: e31896. 10.7759/cureus.31896 PMC9792336 36579250

[48] Sanderson E, Glymour MM, Holmes MV, Kang H, Morrison J, Munafò MR, *et al*. Mendelian randomization. Nature Reviews Methods Primers. 2022; 2: 6. 10.1038/s43586-021-00092-5 PMC7614635 37325194

[49] Yang L, Wang L, Zheng YB, Liu Y, Bao EH, Wang JH, *et al*. Causal relationship between periodontitis and prostate diseases: a bidirectional Mendelian randomization study. Clinical Oral Investigations. 2025; 29: 127. 10.1007/s00784-025-06211-w 39945912

[50] Zhang B, Wang X, Gu Y, Zhang Q, Liu L, Meng G, *et al*. The association between grip strength and incident carotid atherosclerosis in middle-aged and older adults: the TCLSIH cohort study. Maturitas. 2023; 167: 53–59. 10.1016/j.maturitas.2022.09.008 36306667

[51] Park SH, Sugier P, Asgari Y, Karimi M, Kaaks R, Fortner RT, *et al*. Reproductive factors, sex hormone levels, and differentiated thyroid cancer risk: a mendelian randomization study. Thyroid®. 2025; 35: 433–443. 10.1089/thy.2024.0548 40137860

[52] Zhu J, Yuan X, Zhang Y. The potential association between sedentary behaviors and risk of temporomandibular disorders: evidence from Mendelian randomization analysis. Journal of Oral & Facial Pain and Headache. 2024; 38: 91–100. 10.22514/jofph.2024.042 PMC11810678 39800960

[53] Richmond RC, Davey Smith G. Mendelian randomization: concepts and scope. Cold Spring Harbor Perspectives in Medicine. 2022; 12: a040501. 10.1101/cshperspect.a040501 PMC8725623 34426474

