# Supplementary material for: Bidirectional causality between female reproductive traits and temporomandibular disorders
Source: J Oral Facial Pain Headache. 2025 Sep 12;39(3):163–71. doi: 10.22514/jofph.2025.058 (PMC12520435; doi:10.22514/jofph.2025.058)
Supplement: Supplementary file 1 [file Supplementary-Figs.docx]

Supplementary material


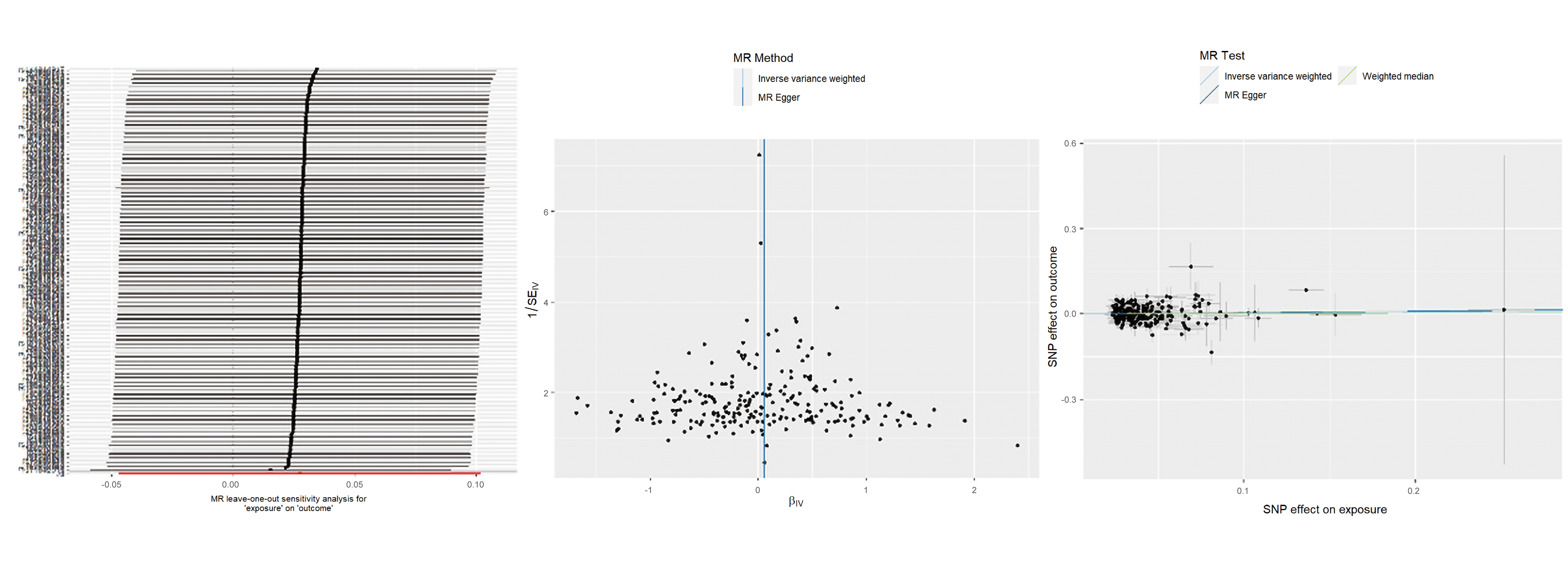


Supplementary Fig. 1. Leave-one-out plots, funnel plots, and scatter plots for AAM as exposure and TMD as outcome. MR: Mendelian randomization; SNP: single-nucleotide polymorphism; SE_IV_: standard error of instrumental variable estimate; Β_IV_: instrumental variable estimate of the causal effect.


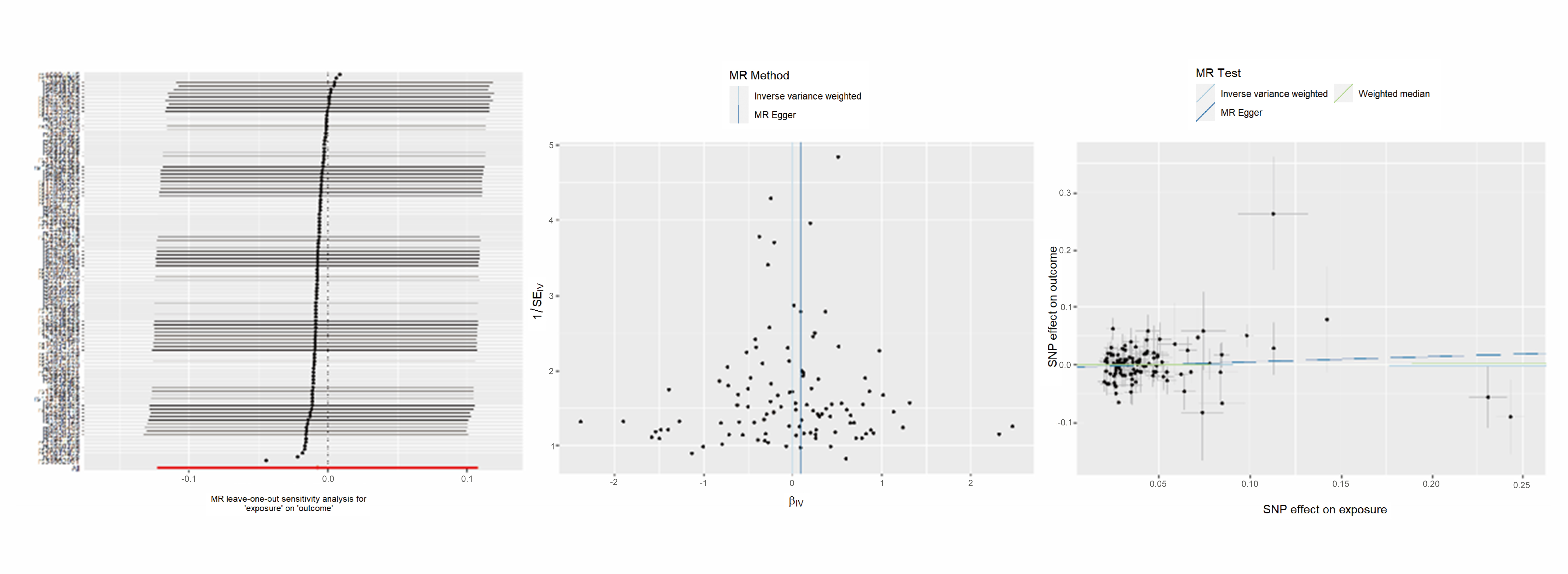


Supplementary Fig. 2. Leave-one-out plots, funnel plots, and scatter plots for ANM as exposure and TMD as outcome. MR: Mendelian randomization; SNP: single-nucleotide polymorphism; SE_IV_: standard error of instrumental variable estimate; Β_IV_: instrumental variable estimate of the causal effect.


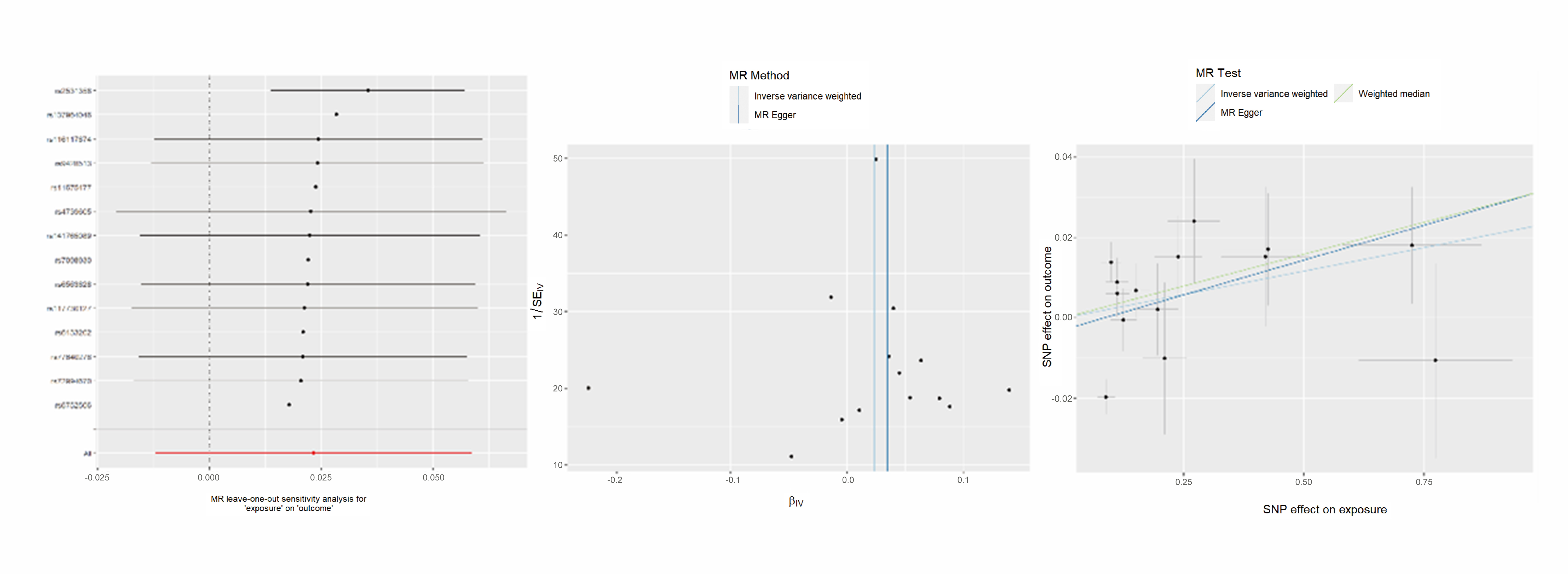


Supplementary Fig. 3. Leave-one-out plots, funnel plots, and scatter plots for TMD as exposure and AAM as outcome. MR: Mendelian randomization; SNP: single-nucleotide polymorphism; SE_IV_: standard error of instrumental variable estimate; Β_IV_: instrumental variable estimate of the causal effect.


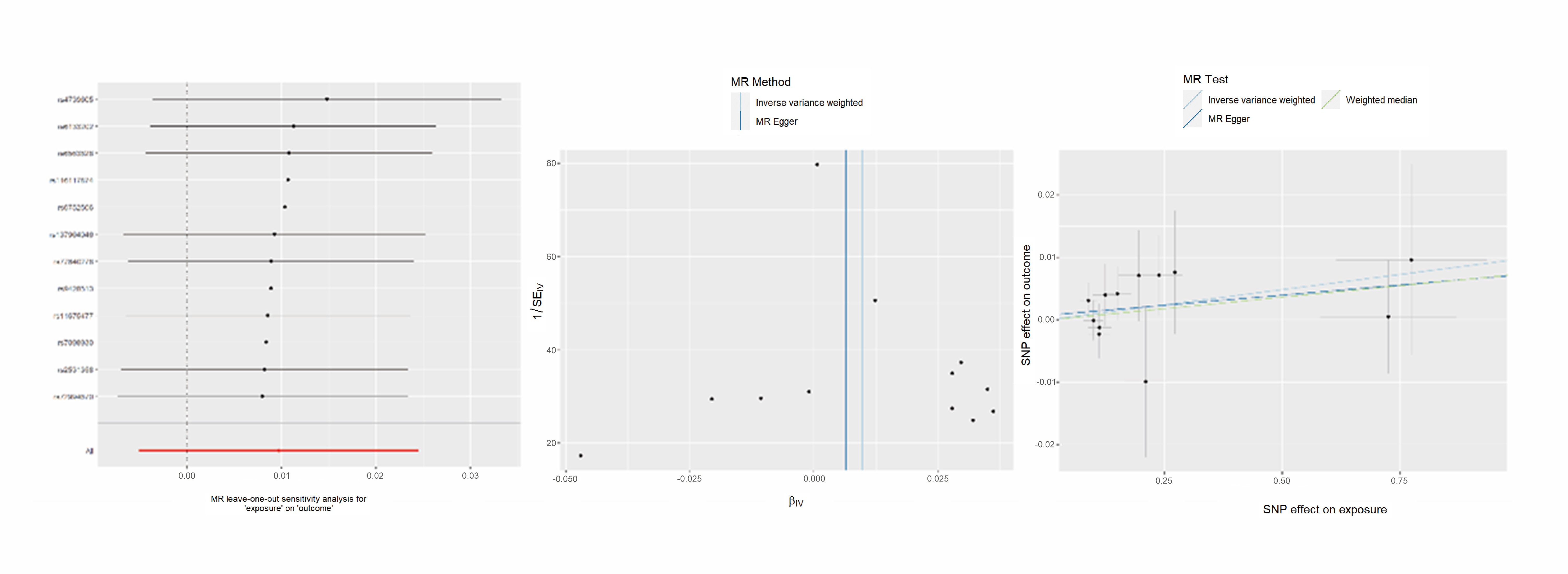


Supplementary Fig. 4. Leave-one-out plots, funnel plots, and scatter plots for TMD as exposure and AFS as outcome. MR: Mendelian randomization; SNP: single-nucleotide polymorphism; SE_IV_: standard error of instrumental variable estimate; Β_IV_: instrumental variable estimate of the causal effect.


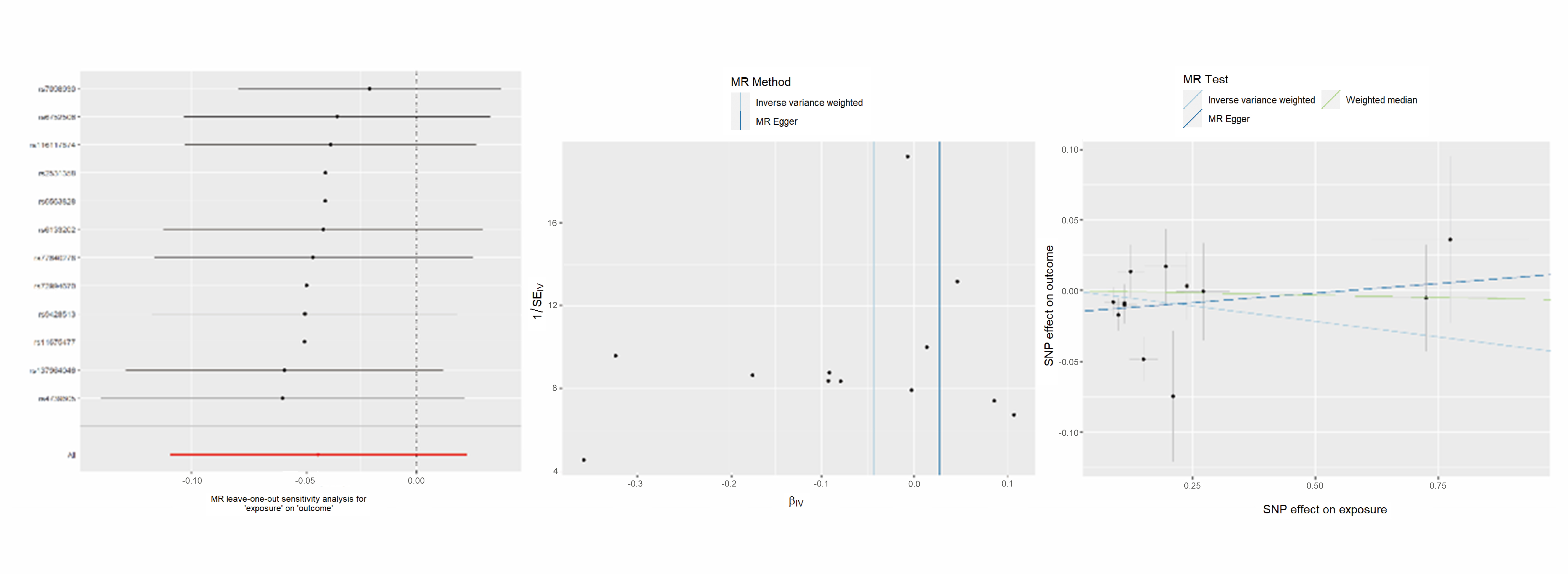


Supplementary Fig. 5. Leave-one-out plots, funnel plots, and scatter plots for TMD as exposure and AFB as outcome. MR: Mendelian randomization; SNP: single-nucleotide polymorphism; SE_IV_: standard error of instrumental variable estimate; Β_IV_: instrumental variable estimate of the causal effect.


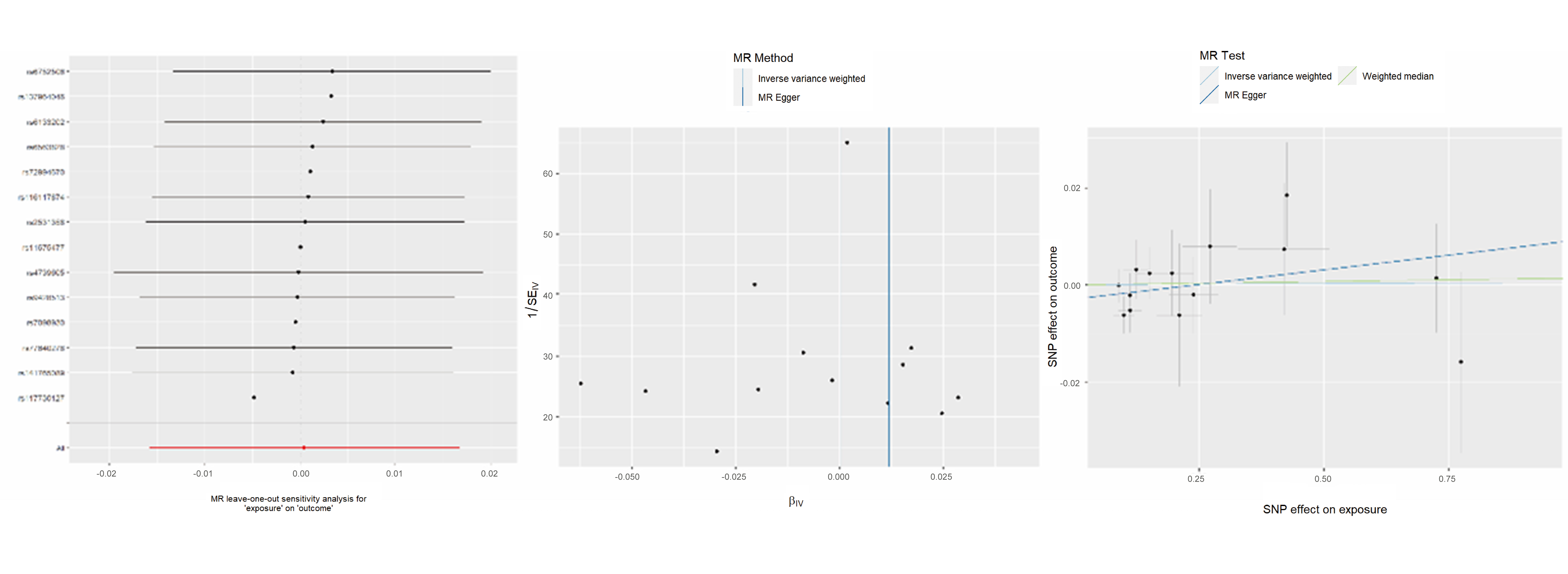


Supplementary Fig. 6. Leave-one-out plots, funnel plots, and scatter plots for TMD as exposure and ALB as outcome. MR: Mendelian randomization; SNP: single-nucleotide polymorphism; SE_IV_: standard error of instrumental variable estimate; Β_IV_: instrumental variable estimate of the causal effect.


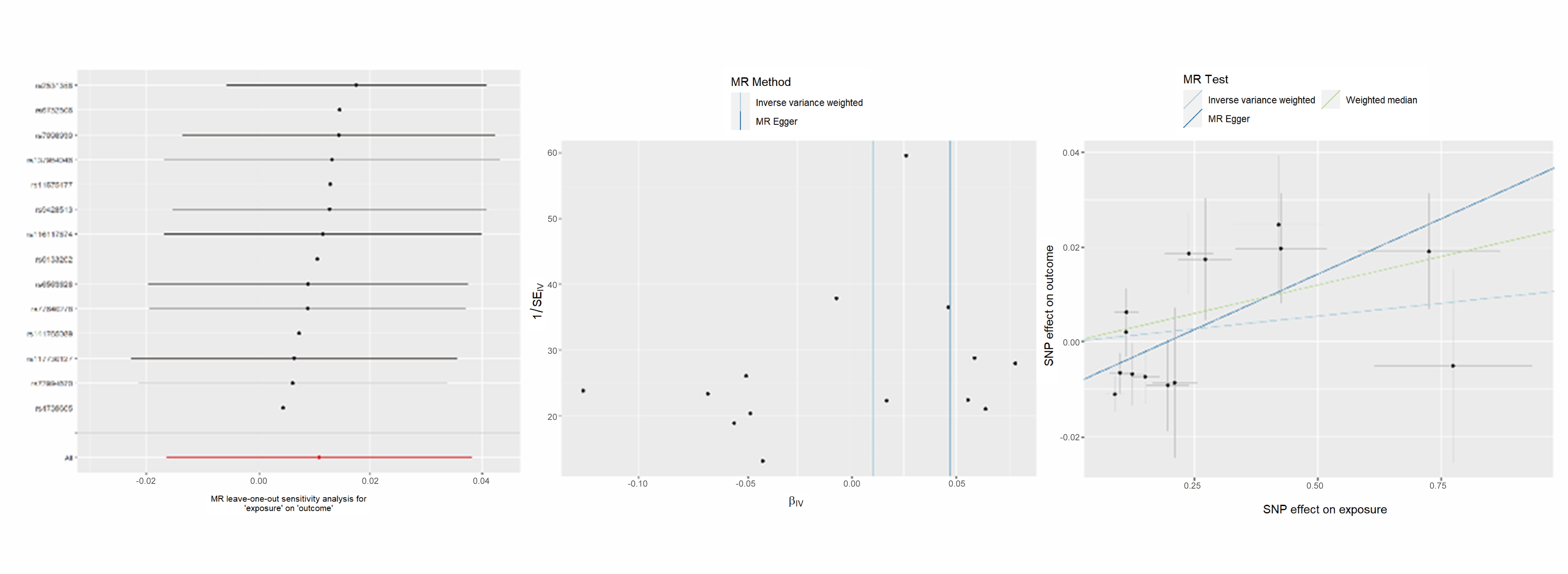


Supplementary Fig. 7. Leave-one-out plots, funnel plots, and scatter plots for TMD as exposure and ANM as outcome. MR: Mendelian randomization; SNP: single-nucleotide polymorphism; SE_IV_: standard error of instrumental variable estimate; Β_IV_: instrumental variable estimate of the causal effect.
