# Supplementary material for: Bidirectional causality between female reproductive traits and temporomandibular disorders
Source: J Oral Facial Pain Headache. 2025 Sep 12;39(3):163–71. doi: 10.22514/jofph.2025.058 (PMC12520435; doi:10.22514/jofph.2025.058)
Supplement: Supplementary file 3 [file Supplementary-Tables.docx]

Supplementary material

Supplementary Table 1. The causal effects of women's reproductive traits on temporomandibular disorders without outliers.

| Exposure | Outcome | nSNP | Methods | OR  (95% CI) | *p*-value | Heterogeneity | | Pleiotropy  *p*-val |
| --- | --- | --- | --- | --- | --- | --- | --- | --- |
|  |  |  |  |  |  | MR Egger  Q_*p*val | IVW  Q_*p*val |  |
| Age at menarche | Temporomandibular disorders | 220 | IVW | 1.03  (0.96–1.10) | 0.458 | 0.068 | 0.074 | 0.779 |
|  |  | 220 | MR Egger | 1.05  (0.87–1.28) | 0.593 |  |  |  |
|  |  | 220 | WM | 1.02  (0.90–1.15) | 0.813 |  |  |  |
| Age at first sexual intercourse | Temporomandibular disorders | 59 | IVW | 0.49  (0.36–0.66) | **2.53 × 10^−6^** | 0.057 | 0.063 | 0.577 |
|  |  | 59 | MR Egger | 0.73  (0.17–3.10) | 0.669 |  |  |  |
|  |  | 59 | WM | 0.58  (0.39–0.86) | **0.007** |  |  |  |
| Age at first sexual intercourse | Temporomandibular disorders | 49 | IVW | 0.89  (0.82–0.98) | **0.015** | 0.118 | 0.123 | 0.421 |
|  |  | 49 | MR Egger | 0.75  (0.49–1.15) | 0.196 |  |  |  |
|  |  | 49 | WM | 0.86  (0.76–0.98) | **0.022** |  |  |  |

IVW: Inverse variance weighted; WM: Weighted median; MR: Mendelian randomization; nSNP: number of single nucleotide polymorphisms; OR: odds ratio; CI: confidence intervals; Q_pval: Cochran’s Q-test *p* value; *p*-val: *p* value. Bold format represents *p* < 0.05 considered statistically different.

Supplementary Table 2. The causal effects of temporomandibular disorders on women's reproductive traits without outliers.

| Exposure | Outcome | nSNP | Methods | OR/Beta  (95% CI) | P-value | Heterogeneity | | Pleiotropy  *p*-val |
| --- | --- | --- | --- | --- | --- | --- | --- | --- |
|  |  |  |  |  |  | MR Egger  Q_*p*val | IVW  Q_*p*val |  |
| Temporomandibular disorders | Age at menarche | 13 | IVW | 0.04  (0.01–0.06) | **0.035** | 0.819 | 0.567 | 0.077 |
|  |  | 13 | MR Egger | 0.01  (−0.02–0.04) | 0.655 |  |  |  |
|  |  | 13 | WM | 0.03  (0.00–0.06) | **0.042** |  |  |  |
| Temporomandibular disorders | Age at natural menopause | 13 | IVW | 0.02  (−0.01–0.04) | 0.137 | 0.140 | 0.082 | 0.164 |
|  |  | 13 | MR Egger | 0.04  (−0.00–0.07) | 0.056 |  |  |  |
|  |  | 13 | WM | 0.02  (−0.01–0.05) | 0.094 |  |  |  |

IVW: Inverse variance weighted; WM: Weighted median; MR: Mendelian randomization; nSNP: number of single nucleotide polymorphisms; Q_*p*val: Cochran’s Q-test *p* value; *p*-val: *p* value. Bold format represents *p* < 0.05 considered statistically different.
